# Melaena with Peutz-Jeghers syndrome: a case report

**DOI:** 10.1186/1752-1947-4-44

**Published:** 2010-02-08

**Authors:** Sayantan Bhattacharya, Sunondo R Mahapatra, Ramlal Nangalia, Amitabh Palit, John R Morrissey, Ernie Ruban, Vijay Jadhav, George Mathew

**Affiliations:** 1 George Eliot Hospital, Nuneaton, CV10 7DJ, UK; 2 Warwick Medical School, University of Warwick, CV4 7AL, UK

## Abstract

**Introduction:**

Peutz-Jeghers syndrome (PJS) is a rare familial disorder characterised by mucocutaneous pigmentation, gastrointestinal and extragastrointestinal hamartomatous polyps and an increased risk of malignancy. Peutz-Jeghers polyps in the bowel may result in intussusception. This complication usually manifests with abdominal pain and signs of intestinal obstruction.

**Case Presentation:**

We report the case of a 24-year-old Caucasian male who presented with melaena. Pigmentation of the buccal mucosa was noted but he was pain-free and examination of the abdomen was unremarkable. Upper gastrointestinal endoscopy revealed multiple polyps. An urgent abdominal computed tomography (CT) scan revealed multiple small bowel intussusceptions. Laparotomy was undertaken on our patient, reducing the intussusceptions and removing the polyps by enterotomies. Bowel resection was not needed.

**Conclusion:**

Melaena in PJS needs to be urgently investigated through a CT scan even in the absence of abdominal pain and when clinical examination of the abdomen shows normal findings. Although rare, the underlying cause could be intussusception, which if missed could result in grave consequences.

## Introduction

Peutz-Jeghers syndrome (PJS) is a rare familial disorder, with an incidence of 1 in 12-30,000 live births [1]. It is an autosomal dominant condition with incomplete penetrance [2]. Nonsense, frameshift and missense mutations inactivating the LKB1 gene on chromosome 19p13.3 have been implicated as the underlying abnormality [3].

PJS presents with characteristic flat, pigmented, freckle-like cutaneous lesions mainly on the lower lip, perioral area, buccal mucosa, periorbital area and eyelids. The syndrome is also associated with gastrointestinal and extragastrointestinal hamartomatous polyps. The typical pathological feature of Peutz-Jegher polyp (PJP) is a smooth muscle core arising from the muscularis mucosae and ramifying into the substance of the polyp like the branches of a tree.

The World Health Organisation (WHO) clinico-pathological criteria for diagnosing this rare disorder are [4]:

1. Three or more polyps, which show histological features consistent with PJS.

2. A family history of PJS with any number of PJPs.

3. A family history of PJS with characteristic mucocutaneous pigmentation.

4. Characteristic mucocutaneous pigmentation with any number of PJPs.

Individuals with this condition carry a very high risk of developing not only gastrointestinal adenocarcinoma but also extra-gastrointestinal malignancies in the breast, pancreas, testes and ovary [5,6]. Compared with the normal population, PJS subjects have a relative risk of 15 for developing any such type of malignancy [6]. Pseudo-invasion, mimicking adenocarcinoma, is described in nearly 10% cases of PJS. It is thought that the mechanical pressure resulting from intussusception of small bowel polyps in PJS may be responsible for misplacing luminal epithelial cells through normal anatomic defects in the intestinal wall, particularly the ones caused by traversing neurovascular bundles [7].

Published review articles can be referenced for further information about this disease [5,8]. Of the many published case reports with solitary or multiple PJPs, [9,10] most patients presented with bleeding and intestinal intussusception [11,12]. To the best of our knowledge, in all those reports of patients with single or multiple enteric intussusception, abdominal signs and symptoms of some kind were present. This led the clinician to suspect the condition is a surgical emergency. Here we report a case of multiple small intestinal intussusception in a young adult man presented with melaena, but with a completely unremarkable abdominal examination.

## Case Presentation

A 24-year-old Caucasian man presented with an acute onset of melaena to the Emergency Department of our hospital. He had no abdominal pain, or history of change in bowel habit or any significant loss of weight or appetite. He was previously healthy and took no regular medications. He had no family history of gastrointestinal disorders. Our patient was also a non-smoker and had no history of illicit drug use.

Upon admission, he was hypotensive and tachycardic. However, abdominal examination was completely unremarkable. Rectal examination revealed black tarry stool. Multiple dark pigmented patches were noted on the buccal mucosa of our patient. Laboratory tests revealed microcytic, hypochromic anaemia with a haemoglobin of 7.6 gm%. Blood urea was elevated at 12 mmol/litre. Initial assessment showed no other abnormalities.

**Figure 1:**
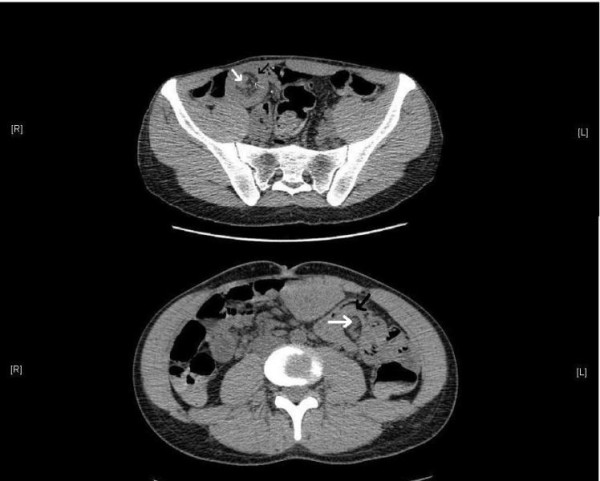
**Axial computed tomography (CT) scan images demonstrating intussusceptions at different levels, with intussuscipiens (*single black arrow*), intussusceptum (*thick white arrow*) and vessels in the invaginated mesenteric fat (*thin white arrow*)**.

He was resuscitated and required transfusion of six units of blood. Urgent upper gastrointestinal endoscopy revealed polyps in the stomach and duodenum, but no active bleeding sites were found. The presence of buccal pigmentation and multiple polyps on endoscopy suggested a diagnosis of PJS. A computed tomography (CT) scan of the abdomen of our patient was therefore performed. This confirmed multiple small bowel intussusceptions. (Figure 1)

**Figure 2:**
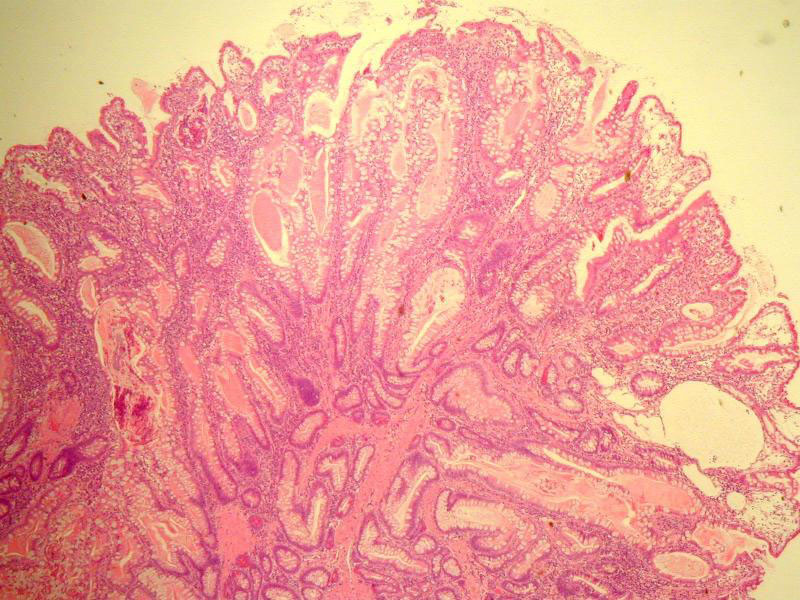
**Histology image, showing a typical Peutz-Jeghers polyp (H and E staining), demonstrating glandular disorganisation, hamartomatous appearance and ramifying branching bundles of smooth muscle**.

Urgent laparotomy was undertaken on our patient and the intussusceptions were reduced. Through enterotomies, eight polyps were removed from the affected segment of the gut. The affected segment of gut was found to be viable. He had a fairly uneventful recovery with no further bleeding. Pathological examination of the polyps confirmed hamartomas with smooth muscle arborisation, compatible with Peutz Jeghers polyps. No features of pseudo-invasion were noticed in any of the polyps. (Figure 2)

## Discussion

Enteric intussusception in adults is very rare and more than 90% of cases are associated with a pathological leading point. In the study conducted by Chiang and Lin, nearly 18% of incidences of hamartomas caused enteric intussusception. Ileo-ileal intussusception was the most common type. About 90% of cases presented with abdominal pain, while 40% exhibited signs of proximal small bowel obstruction. Rare presentations were diarrhoea, bleeding and anaemia. Abdominal CT scan was shown to be the most effective diagnostic instrument [13].

Our patient presented to the Emergency Department with melaena, and on clinical examination the abdomen was completely unremarkable. Since the presentation was that of upper gastrointestinal bleeding, initial management would normally be conservative, followed by endoscopy and further intervention according to need [14]. However, in the rare situation when bleeding is the result of small bowel intussusceptions, such a conservative approach could result in bowel ischaemia. In this case, the suspicion of PJS led to active investigation by a CT scan, thereby revealing the intussusceptions.

The CT scans obtained in this case showed presence of several ileo-ileal intussusceptions. (Figure 1)

Management of melaena from non-variceal causes has been extensively reviewed in the literature [15]. Prognosis in terms of rebleeding, length of stay in hospital and need for further endoscopic or surgical intervention can be assessed by Rockall [16] and Blatchford scores [17] (Table S1, Additional file 1 and Table S2, Additional file 2). Our patient had a Rockall score of 3 with an 11.2% risk of rebleeding and a Blatchford score of 14, indicating a high likelihood of the need for further intervention.

It has been widely debated whether reduction of intussusception should precede resection. Reduction of large bowel intussusceptions runs the risk of perforation and contamination of the peritoneal cavity with faeces or tumour cells (when the lead point is a tumour, more commonly found in large bowel than in small bowel intussusceptions in adults). Therefore, en bloc resection before reduction is advocated with large bowel intussusceptions, whereas reduction may be attempted in small intestine [18,19]. In our patient, the intussusceptions were entirely small intestinal and were successfully reduced through surgery. The polyps were removed by enterotomies. No resection was needed.

## Conclusion

We conclude that melaena in a case of PJS may indicate an underlying surgical emergency like intussusception, which if missed can result into bowel ischaemia with grave consequences. Though a series of similar cases would be needed to formulate a generalised treatment plan for all such cases in this category, the significance of an urgent abdominal imaging to exclude a surgical pathology is well-reflected in our case report.

## Abbreviations

CT: Computed Tomography; PJS: Peutz-Jeghers Syndrome; PJP: Peutz-Jeghers polyps.

## Consent

Written informed consent was obtained from the patient for publication of this case and any accompanying images. A copy of the written consent is available for review by the Editor-in-Chief of this journal.

## Competing interests

The authors declare that they have no competing interests.

## Authors' contributions

SB and SM prepared the manuscript and got the consent of the patient. RN, AP, VJ, GM and JRM independently reviewed the manuscript and made corrections. AP contributed the radiology images and ER contributed the histology images. All authors have read and approved the final version of the manuscript.

## Supplementary Material

Additional file 1 **Table S1**. Rockall Score. Click here for file

Additional file 2 **Table S2**. Blatchford Score - Scoring system identifying patients with upper gastrointestinal bleed, who would need intervention. Click here for file
