# Supplementary figures and images for: Melaena with Peutz-Jeghers syndrome: a case report
Source: J Med Case Rep. 2010 Feb 8;4:44. doi: 10.1186/1752-1947-4-44 (PMC2830979; doi:10.1186/1752-1947-4-44)

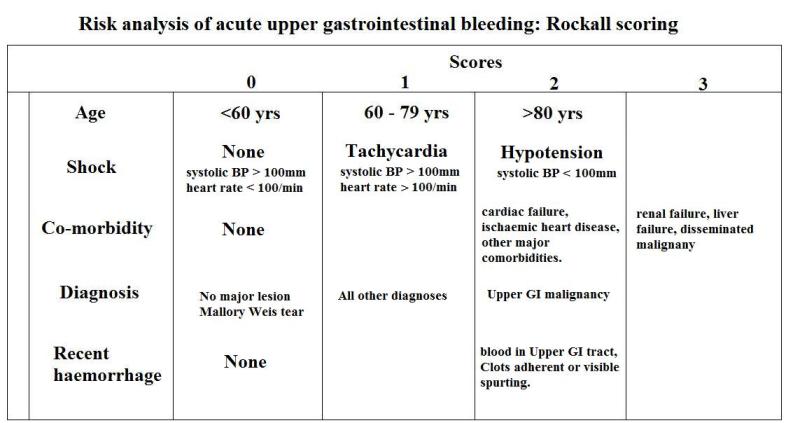

Supplement: Additional file 1 — Table S1. Rockall Score. [file 1752-1947-4-44-S1.JPEG]

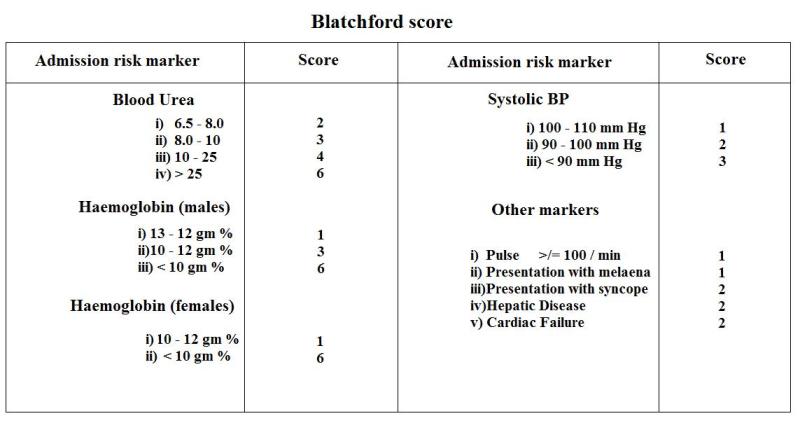

Supplement: Additional file 2 — Table S2. Blatchford Score - Scoring system identifying patients with upper gastrointestinal bleed, who would need intervention. [file 1752-1947-4-44-S2.JPEG]
